# Faraday rotation and photoluminescence in heavily Tb^3+^-doped GeO_2_-B_2_O_3_-Al_2_O_3_-Ga_2_O_3_ glasses for fiber-integrated magneto-optics

**DOI:** 10.1038/srep08942

**Published:** 2015-03-10

**Authors:** Guojun Gao, Anja Winterstein-Beckmann, Oleksii Surzhenko, Carsten Dubs, Jan Dellith, Markus A. Schmidt, Lothar Wondraczek

**Affiliations:** 1 Otto Schott Institute of Materials Research, University of Jena, 07743 Jena, Germany; 2 Innovent e.V., 07745 Jena, Germany; 3 Leibniz Institute of Photonic Technology, 07745 Jena, Germany

## Abstract

We report on the magneto-optical (MO) properties of heavily Tb^3+^-doped GeO_2_-B_2_O_3_-Al_2_O_3_-Ga_2_O_3_ glasses towards fiber-integrated paramagnetic MO devices. For a Tb^3+^ ion concentration of up to 9.7 × 10^21^ cm^−3^, the reported glass exhibits an absolute negative Faraday rotation of ~120 rad/T/m at 632.8 nm. The optimum spectral ratio between Verdet constant and light transmittance over the spectral window of 400–1500 nm is found for a Tb^3+^ concentration of ~6.5 × 10^21^ cm^−3^. For this glass, the crystallization stability, expressed as the difference between glass transition temperature and onset temperature of melt crystallization exceeds 100 K, which is a prerequisite for fiber drawing. In addition, a high activation energy of crystallization is achieved at this composition. Optical absorption occurs in the NUV and blue spectral region, accompanied by Tb^3+^ photoluminescence. In the heavily doped materials, a UV/blue-to-green photo-conversion gain of ~43% is achieved. The lifetime of photoluminescence is ~2.2 ms at a stimulated emission cross-section *σ*_em_ of ~1.1 × 10^−21^ cm^2^ for ~ 5.0 × 10^21^ cm^−3^ Tb^3+^. This results in an optical gain parameter *σ*_em_*τ of ~2.5 × 10^−24^ cm^2^s, what could be of interest for implementation of a Tb^3+^ fiber laser.

The Faraday effect reflects the ability of a material to - in the presence of a magnetic field being parallel to the incident light beam - rotate the polarization plane of linear polarized light by a certain angle^1^,^2^,^3^. The material's magneto-optical (MO) performance is typically described by the Verdet constant *V*_B_, which represents the degree of rotation as a function of the acting magnetic field strength and the geometrical path length within the material. High performance can hence be achieved via large rotation efficiency or a long path length. Applications of MO materials range from magnetic field sensing and security encoding to optical modulators, diodes, isolators and switches^1^,^2^,^3^,^4^,^5^,^6^. Key for the design of an efficient, optically transparent (bulk) MO material is the incorporation of a high atom concentration of paramagnetic species while, at the same time, avoiding optical absorption to the highest possible degree. While some transition metals have also been considered for this purpose, at present, this calls for the use of rare earth species^7^. Here, due to the electronic transition of 4f^8^ → 4f^7^5d^8^,^9^, the Tb^3+^ ion offers one of the highest paramagnetic susceptibilities (*J* = 6, *g* = 1.46) and magnetic moments (9.5–9.72 μ_eff_) of all rare earth ions. Consequently, the most promising bulk MO material is terbium aluminum garnet (Tb_3_Al_5_O_13_, TAG, *V*_B_ ~ 180 rad/T/m)^10^,^11^, which is not yet available commercially, though. Instead, terbium gallium garnet single crystals (Tb_3_Ga_5_O_13_, TGG, *V*_B_ ~ 134 rad/T/m)^12^ are presently the most widely used commercial MO materials. But also all commercially available MO glasses rely on massive Tb^3+^-doping^4^,^13^,^14^,^15^,^16^. As an alternative to the MO crystals, glassy materials offer a much improved flexibility of forming and processing. Especially glass compositions which are suitable for fiber fabrication could enable fiber-integrated devices. In addition, the higher interaction length which can be achieved in fiber devices could further compensate eventual losses in Faraday rotation efficiency. In this regard, besides the primary optical properties, the thermo-physical stability and the rheological properties of the considered glass and its corresponding (supercooled) melt are key parameters: in order to avoid crystallization of the melt during fiber drawing, a certain crystallization stability is required. This is often expressed as the difference, Δ*T*, between the glass transition temperature *T*_g_ and the onset temperature of crystallization *T*_c_, or through various other empirical stability indicators such as the Hrubý parameter which is derived from this difference, sometimes further relating it to the liquidus temperature of the melt or other properties^17^,^18^. Typically, a large value of Δ*T* is sought for two reasons: fiber drawing must be performed at a temperature sufficiently above *T*_g_ so that a sufficiently low viscosity is reached and the interval of processing temperature must be sufficiently wide to tolerate a certain degree of processing-induced temperature variability. On the other hand, for many of the specialty (non-silica) compositions with often high liquid fragility, fiber drawing cannot be performed above the liquidus temperature (where there would not be any risk of crystallization) because then, the viscosity would be too low.

Here, we consider glass forming liquids of the type GeO_2_-B_2_O_3_-Al_2_O_3_-Ga_2_O_3_ enabling high rare earth solubility. In this system, we achieve a Tb_2_O_3_ doping concentration of up to 25 mol%. The glass stability parameters are controlled through tailoring the matrix composition in order to provide the possibility of fiber drawing. We then report on the MO and photoluminescence properties of this material.

## Results

### Magneto-optical properties

**Figure 1:**
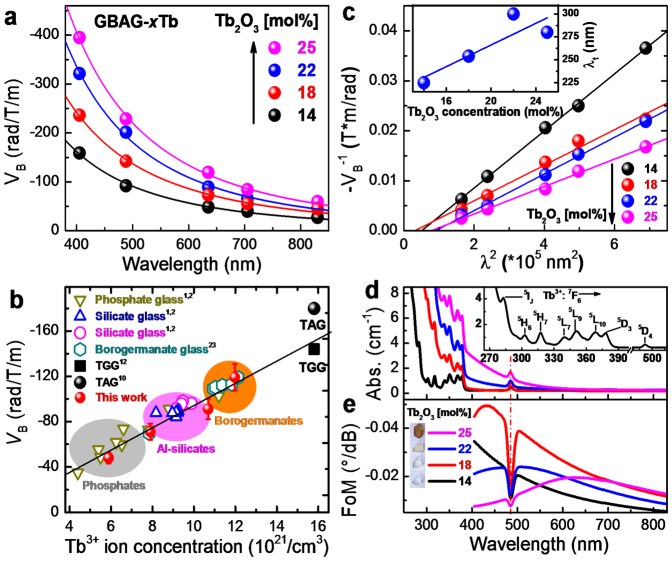
Magneto-optical properties of GBAG-*x*Tb glasses. (a) Variation of the Verdet constant with wavelength for GBAG-*x*Tb glasses as a function of Tb_2_O_3_ concentration at room-temperature. The solid lines represent a fit of the data to the power function *y* = *a*(1-*x*)*^b^*. (b) Dependence of *V*_B_ on Tb^3+^ ion concentration and comparison to other reported data glasses at a fixed wavelength of 632.8 nm. (c) Van Vleck-plot of the inverse *V*_B_ (*V*_B_^−1^) over the square wavelength (*λ*^2^). The solid lines in (c) represents a linear fit of the data. The inset of (c) shows the value of the transition wavelength *λ*_t_ versus Tb_2_O_3_ concentration. In (d), the UV-VIS-NIR optical absorption spectra are given, from which the spectral MO figure of merit is obtained (shown in (e)). The inset of (d) exemplarily shows a zoom at the absorption spectrum in the spectral region of 260–550 nm for GBAG-14Tb.

The chemical composition and physical properties of all samples are summarized in Tables 1–2. Figure 1a shows the room-temperature wavelength dependence of *V*_B_ for the full series of GBAG-*x*Tb (*x* = 14, 18, 22 and 25). As expected, all samples exhibit paramagnetic behaviour over the full range of studied wavelengths, with a strong absolute increase towards the blue. Secondly, there is a notable increase with increasing Tb^3+^ dopant concentration, i.e., from ~48 to 119 rad/T/m at 632.8 nm (Fig. 1a and 1b). For similar Tb^3+^ ion concentration, the absolute value *V*_B_ of GBAG-*x*Tb glasses is in the order of that of other reported record values in Tb^3+^-doped MO glasses, e.g, silicate^2^,^6^, phosphate^2^, borate^5^,^22^ and borogermanate^23^ glasses (Fig. 1b). For the maximum Tb^3+^ loading we report here, GBAG-25Tb, *V*_B_ exceeds the rotation efficiency in most of the well-known MO glasses, *e.g.*, 30Tb_2_O_3_-70B_2_O_3_ (~103 rad/T/m)^22^ and 25Tb_2_O_3_-15Al_2_O_3_-60SiO_2_ (~102 rad/T/m)^6^, and is similar with that of 33Tb_2_O_3_-25GeO_2_-25B_2_O_3_-5SiO_2_-12Al_2_O_3_ (~119 rad/T/m)^23^. For comparison, data for the single crystalline benchmarks of TAG and TGG are also shown in Fig. 1b^11^,^12^.

In the framework of the Van Vleck-Hebb model of single-oscillator paramagnetic rare earth ions, the relationship between V and λ^2^ can be written as^24^,^25^
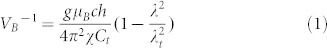
In Eq. (1), *g* is the Landé factor, *c* the velocity of light, *h* is the Planck constant, *C*_t_ is the effective transition probability, and *λ*_t_ is the effective transition wavelength. *λ*_t_ is a weighted average value which is taken as the origin of the paramagnetic Faraday rotation. In rare earth ions, it is close to the position of the electric transition of 4f^n^ ↔ 4f^n−1^5d^26^. Plotting *V*^−1^ over *λ*^2^ therefore yields a linear relationship (Fig. 1c). Here, *λ*_t_ is the intersection with the λ^2^ axis which results from extrapolation of the data. The value of *λ*_t_ is dependent on Tb_2_O_3_ concentration (inset of Fig. 1c). It increases with Tb_2_O_3_ concentration, i.e., from ~225 to 300 nm when *x* ≤ 22. A decrease back to ~280 nm is observed for the highest Tb_2_O_3_ concentration. As expected, these values are close to the 4f^8^ ↔ 4f^7^5d transition of the Tb^3+^ ion (~250 nm)^5^, and are also similar to other reported values, *e.g.*, Tb^3+^-doped phosphate (~250 nm)^27^, borosilicate (~259–280 nm)^28^, aluminoborate (~250 nm)^24^, sodium borate (~220 nm)^8^ and fluorophosphate glasses (~217 nm)^26^.

**Figure 2:**
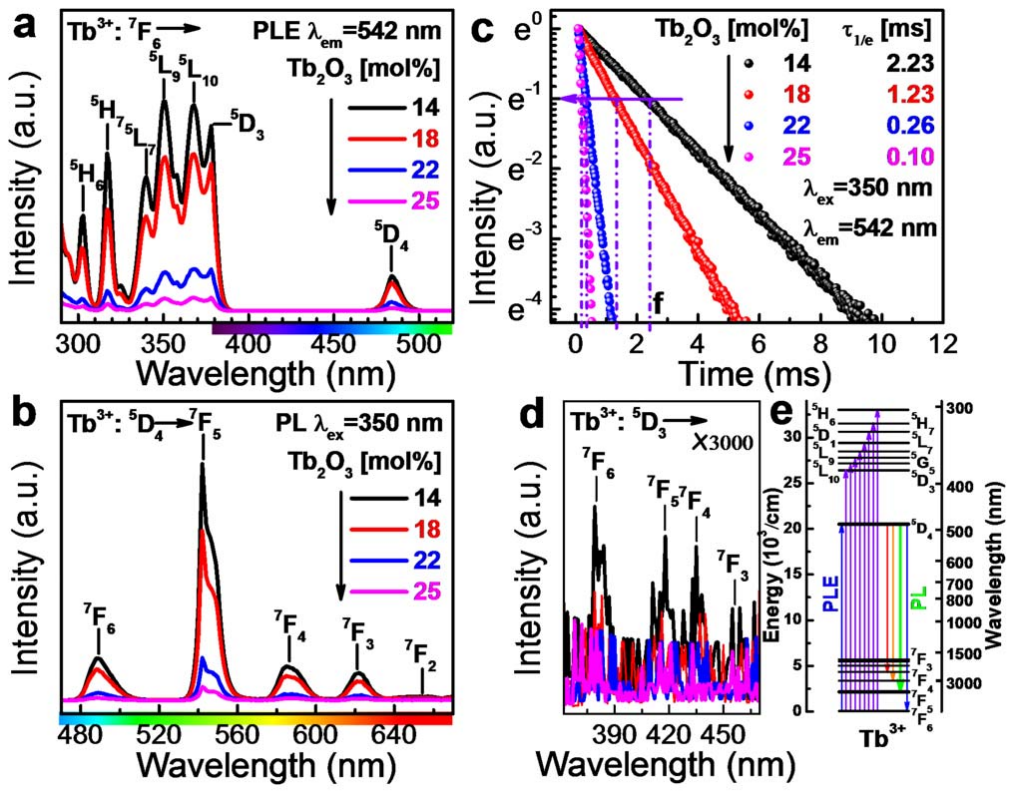
Photoluminescence of GBAG-*x*Tb glasses. Static (a) PLE and (b) PL spectra, and (c) normalized dynamic decay curves of photoluminescence from GBAG-*x*Tb as a function of Tb_2_O_3_ concentration at room-temperature. (d) is a zoom (by a factor of 3000) into the PLE spectra at the spectra region of 360–470 nm. (e) represents the energy level diagram of Tb^3+^. The labels in (a–b) indicate the respective band assignment.

Fig. 1d shows the UV- NIR optical absorption spectra of GBAG-*x*Tb (*x* = 14, 18, 22 and 25). The absorption spectra consist of several strongly overlapping but sharp absorption bands in the 300 to 390 nm range, and another sharp band at ~484 nm. These bands can readily be assigned to the 4f^8^ → 4f^8^ electronic transitions of Tb^3+^ from the ground state of ^7^F_6_ to the labeled excited states (inset of Fig. 1d and Fig. 2e)^29^,^30^. The intensity of all bands follows well Lambert-Beer's power law. All glasses exhibit high transparency in the ~400 to 1500 nm range with a transmittance of ~58% (~95%) with a thickness of 1 cm (mm). The increasing absorption intensity with increasing Tb^3+^ content in the near-UV region results in a shift of the absorption edge and an apparent coloration under sunlight, gradually varying from colorless to brown (inset of Fig. 1e).

The MO figure of merit (FoM) which is an important parameter for practical applications results from the ratio of *V*_B_/*a*, where *a* is absorption coefficient^4^. As displayed in Fig. 1e, the spectral FoM exhibits a sharp dip at 484 nm, resulting from the ^7^F_6_ → ^5^D_4_ absorption band of Tb^3+^. In the present case, the glass of GBAG-18Tb exhibits the best trade-off between *V*_B_ and *a* over the whole spectrum. The highest FoM performance of ~−0.049°/dB is found at ~435 nm, which matches the emission characteristics of various blue laser diodes.

### Photoluminescence properties

Fig. 2a and 2b present static photoexcitation (PLE) and luminescence (PL) spectra of Tb^3+^ in GBAG-*x*Tb (*x* = 14, 18, 22 and 25) at room temperature. Fully consistent with the optical absorption data (Fig. 1d), the PLE spectra of Tb^3+^ consist of a series of sharp overlapping PLE bands in the NUV region with maxima at 378, 368, 358, 350, 340, 325, 317 and 303 nm, and another sharp PLE line in the blue with a maximum at 484 nm. These bands are attributed to the intra-configurational parity-forbidden 4f^8^ → 4f^8^ electronic transitions from the ground state ^7^F_6_ to the labeled excited states, also indicated in energy level diagram of Tb^3+^ (Fig. 2a and 2e)^30^,^31^,^32^,^33^,^34^. The strongest PLE band is the ^7^F_6_ → ^5^L_9_ at 350 nm, used in the following as excitation wavelength to record the PL spectra. Here, the five typical PL bands of Tb^3+^ are observed, i.e., at 488, 542, 585, 622 and 655 nm, deriving again from the intra-configurational parity-forbidden 4f^8^ → 4f^8^ transitions from ^5^D_4_ to the ^7^F_J__(J = 6, 5, 4, 3 and 2)_ multiplet, Fig. 2b and 2e^35^. The green PL line of the magnetic dipole allowed transition (Δ*J* = 1) Tb^3+^: ^5^D_4_ → ^7^F_5_ at 542 nm with a full width at half maximum (FWHM) of ~10 nm (~337 cm^−1^) dominates the PL spectra for all samples. As a result, the corresponding International Commission on Illumination (CIE) 1931 PL chromaticity coordinates of all samples are (~0.344 ± 0.002, ~0.592 ± 0.002), which are located in the green region. The aforementioned green PL band is Stark-split into two peaks due to the distorting effect of the disordered glass network on the Tb^3+^ ions (Fig. 2b). For lower amounts of Tb_2_O_3_ loading (*x* ≤ 18), the intensity of all PL lines of Tb^3+^ decreases only slightly with Tb_2_O_3_ concentration while for *x* ≥ 18, we observe strong concentration quenching^36^,^37^. This is related to an increasing probability for the formation of Tb-O-Tb entities in the first coordination shell of Tb^3+^.^38^ The concentration quenching effect is further confirmed by the decay data of the Tb^3+^: ^5^D_4_ → ^7^F_5_ emission (Fig. 2c). All decay curves follow a single exponential function of the form *I* = *I*_0_exp(-*t*/*τ*) (with time *t* and intensity *I*). The effective lifetime *τ* decreases with increasing Tb^3+^ concentration, i.e., from ~2.2 to ~1.2 ms when *x* ≤ 18, and further to ~0.1 ms for *x* > 18 (Fig. 2b and 2c). The lifetime of Tb^3+^: ^5^D_4_ → ^7^F_5_ PL for GBAG-14Tb glass (~2.23 ms) is larger than what is found in similar B_2_O_3_-GeO_2_-Gd_2_O_3_ glasses before concentration quenching, ~1.80 ms^39^. This further indicates that particularly weak concentration quenching occurs in the present case. The internal quantum efficiency *η*_iQE_ for GBAG-14Tb is ~63%. This value is close to the highest reported *η*_iQE_ of Tb^3+^-based PL in literature, *e.g.*, Tb^3+^-doped phosphate glass (~78%)^40^ and silicone hybrid materials (~68%)^41^. For higher doping concentration, it decreases to only 0.3% at *x* = 25. The high Tb^3+^ loading results in a large absorption cross-section of incoming light and, hence, high photo-conversion gain. That is, the absorbance of GBAG-14Tb at 350 nm is ~69%. Thus, the external quantum efficiency *η*_eQE_ is ~43%, meaning that at the considered excitation wavelength, ~43% of the incoming photons are converted through photoluminescence.

PL of Tb^3+^ from the higher excited states, *i.e.*, ^5^D_3_ → ^7^F_J__(J = 6, 5, 4, 3, 2 and 1)_ is almost fully quenched even in GBAG-14Tb (Fig. 2d). This is a result of the strong cross-relaxation processes which occur at the high doping levels used in this study. The cross relaxation process is caused by the closeness of the ^5^D_3_ and ^5^D_4_ (~5629 cm^−1^), and the ^7^F_6_ and ^7^F_0_ energy levels (~5791 cm^−1^, Fig. 2e)^42^,
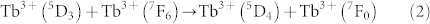


The absorption cross-section *σ*_abs_ of Tb^3+^ at 350 (Tb^3+^: ^7^F_6_ → ^5^L_9_) and 484 nm (Tb^3+^: ^5^F_6_ → ^7^D_4_), and the stimulated emission cross-section σ_em_ of Tb^3+^ PL at 542 nm (Tb^3+^: ^5^D_4_ → ^7^F_5_) can be estimated through McCumber's and Füchtbauer-Ladenburg's equation^43^,^44^,^45^,
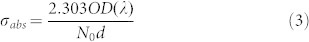

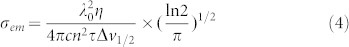
In Eqs. (3)–(4), *N*_0_ is the ion concentration of Tb^3+^, *d* is the sample thickness, *λ*_0_ is the emission wavelength, *OD*(*λ*) is the optical density, *η* is the internal quantum efficiency, *n* is the refractive index of the host material, *τ* is the emission lifetime and Δν_1/2_ is the FWHM of the transition. The *σ*_abs_ value of Tb^3+^ in GBAG-*x*Tb glasses at 350 and 484 nm is calculated to be ~4.94 and 0.95 × 10^−22^ cm^2^, respectively. This value is comparable to that of phosphate glasses (~1.1 × 10^−22^ cm^2^)^40^. The *σ*_em_ value of Tb^3+^ in GBAG-*x*Tb glasses at 542 nm is ~1.1 × 10^−21^ cm^2^, notably larger than in phosphate glasses (~7.4 × 10^−22^ cm^2^)^40^. The product of *σ*_em_*τ, the optical gain parameter for laser applications, is proportional to the amplification gain and inverse laser oscillation threshold^45^. A relatively high value of ~2.5 × 10^−24^ cm^2^s is obtained for the GBAG-14Tb glass, what suggests a large amplification gain and low oscillation threshold and, hence, potential interest for further examination as a green laser gain material.

### Thermal properties

**Figure 3:**
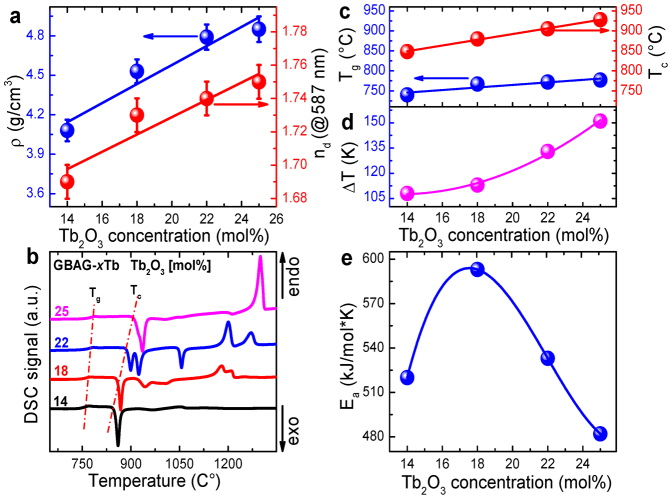
Thermal properties of GBAG-*x*Tb glasses. (a) Physical properties density and refractive index of GBAG-*x*Tb as a function of Tb_2_O_3_ concentration. (b) DSC curves of GBAG-*x*Tb as dependent on Tb_2_O_3_ concentration. From (b), the variation of the glass transition temperature *T*_g_ and the onset temperature of crystallization *T*_c_ are extracted (c). (d–e) show the glass stability parameter Δ*T* and the apparent activation energy of crystallization, respectively, of GBAG-*x*Tb as a function of Tb_2_O_3_ concentration. Solid lines are drawn as guides for the eye.

The values of *ρ, n*_d_, *T*_g_, *T*_c_ and Δ*T* of GBAG-*x*Tb are summarized in Tab. 1. Density and refractive index increase from ~4.08 to 4.85 g/cm^3^ and from 1.69 to 1.75 respectively with increasing Tb_2_O_3_ concentration due to the much higher molar mass of Tb_2_O_3_ (365.85 g/mol) as compared to Ga_2_O_3_ (187.44 g/mol) (Fig. 3a).

Figure 3b shows DSC curves of GBAG-*x*Tb. Here, *T*_g_ and *T*_c_ gradually increase from 740 to 777°C and from 848 to 928°C, respectively, with increasing Tb_2_O_3_ concentration (Fig. 3c). In order to empirically judge glass stability, Δ*T* = *T*_c_–*T*_g_ is calculated from these data.

Generally speaking, larger values of Δ*T* reflect an improved stability against crystallization. Here, Δ*T* increases from 108 to 151 K with increasing of Tb_2_O_3_ concentration (Fig. 3d). Overall, this suggests a comparably high crystallization stability of the glasses of this study.

The apparent activation energy *E*_a_ of crystallization is calculated from the DSC data for varying heating rates by a Kissinger equation^46^,
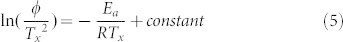
In Eq. (5), *R* is the ideal gas constant, *T*_*x*_ is the temperature of crystallization, and *ϕ* is the heating rate of the DSC experiment. *E*_a_ can therefore be estimated from the slope of a linear fit of ln(ϕ/*T*_x_^2^) versus 1/*T*_x_ plot. The obtained value depends on Tb_2_O_3_ concentration. It reaches a maximum of ~593 kJ/(mol × K) at GBAG-18Tb and decreases to 482 kJ/(mol × K) for *x* = 25 (Fig. 3e). Hence, while, GBAG-18Tb and GBAG-14Tb exhibit the highest MO FoM and the highest PL performance, they also exhibit large Δ*T* and comparatively high *E*_a_.

## Conclusions

In summary, we reported on the magneto-optical (MO) properties of heavily Tb^3+^-doped GeO_2_-B_2_O_3_-Al_2_O_3_-Ga_2_O_3_ glasses towards fiber-integrated paramagnetic MO devices. For Tb^3+^ ion concentrations of up to 9.7 × 10^21^ cm^−3^, the reported glass exhibits an absolute negative Faraday rotation of ~120 rad/T/m at 632.8 nm. The underlying effective transition wavelength *λ*_t_ is close to the 4f^8^ ↔ 4f^7^5d transition of the Tb^3+^ ion, ~250 nm. The optimum FoM is found for a Tb^3+^ concentration of ~6.5 × 10^21^ cm^−3^ (GBAG-18Tb), ~−0.05°/dB at ~435 nm, matching the emission characteristics of blue light-emitting diodes. For this glass, the crystallization stability, expressed as the difference between glass transition temperature and onset temperature of melt crystallization exceeds 100 K, which is a prerequisite for fiber drawing. In addition, a high activation energy of crystallization is achieved using this composition. Optical absorption occurs in the NUV and blue spectral region, accompanied by Tb^3+^ photoluminescence. In the heavily doped materials, a UV/blue-to-green photo-conversion gain of ~43% is achieved. The Tb^3+^ ions are well dispersed in GBAG-*x*Tb glasses without notable concentration quenching of photoluminescence up to a dopant concentration of ~14 mol% of Tb_2_O_3_ (GBAG-14Tb). The lifetime of photoluminescence is ~2.2 ms with a stimulated emission cross-section *σ*_em_ of ~1.1 × 10^−21^ cm^2^ for ~5.0 × 10^21^ cm^−3^ Tb^3+^. This results in an optical gain parameter *σ*_em_*τ of ~2.5 × 10^−24^ cm^2^s, what could be of interest for implementation of a Tb^3+^ fiber laser.

## Methods

### Synthesis of Glasses

Precursor glasses with nominal compositions of 16.5GeO_2_-21.5B_2_O_3_-37Al_2_O_3_-(25–*x*)Ga_2_O_3_-*x*Tb_2_O_3_ (GBAG-*x*Tb with *x* = 14, 18, 22 and 25 mol%) were prepared by conventional melting and quenching. Batches of ~50 g of GeO_2_ (99.99%), H_3_BO_3_ (99.99%), Ga_2_O_3_ (99.99%) and Tb_4_O_7_ (99.99%) were thoroughly mixed and melted in a resistive heating furnace at 1500°C for 3 h in Al_2_O_3_ crucibles, heating to 700°C at 5 K/min and to 1500°C at 10 K/min. Melting conditions were kept identical for all batches to ensure a homogenous dilution of Al_2_O_3_ in the melt. Subsequently, melts were poured onto preheated brass plates, annealed for 1 h and finally cooled down to room temperature at the intrinsic furnace rate (~1 K/min). The obtained glass slabs were cut and polished on both sides for optical characterization.

### Magneto-optical properties

Frequency-dependent MO analyses were done by using a series of laser diodes as light sources (405, 488, 635, 705 and 830 nm) and fitting the obtained data of Faraday rotation to a power function of the form *V_B_* = a(1-*λ*)^b^ with wavelength *λ*. *V*_B_ is calculated from the Faraday rotation angle *θ*_F_, the strength of the external magnetic field *B*, and the length of the light path *L* in the sample, *V*_B_ = *θ*_F_/*BL*.^5^,^8^,^19^ For this, the rotation of the polarization plane was measured with a polarimeter (PAX570VIST/PAX570IR-1T). In this set-up, the magnetic field was applied through a permanent magnet, achieving a constant magnetic flux of 0.23 T. In a second set of experiments, the value if *V*_B_ was derived from the *θ*_F_ versus B dependencies in an iron-yoke magnet (−0.1 T < B < 0.1 T), using a light-emitting diode with the central wavelength of 625 nm. For this, the magnetic flux was swept in the given range with a step-width of ~1 mT. Then, the obtained data on *θ*_F_ versus H were linearly extrapolated to obtain the slope d*θ*_F_/dH, which was used to estimate *V_B_*. Error bars on the value of *V* were obtained from the comparison of those two experiments. UV-VIS-NIR absorption spectra were recorded over the spectral range of 200 nm to 2500 nm in a UV-NIR spectrophotometer (Perkin Elmer, Lambda 950).

### Photoluminescence properties

Static photoexcitation (PLE) and luminescence (PL) spectra and dynamic decay curves of the Tb^3+^-related photoluminescence were recorded with a high-resolution spectrofluorometer (Horiba Jobin Yvon Fluorolog FL3-22) at room temperature. PLE spectra were corrected over the lamp intensity with a silicon photodiode. PL spectra were corrected by the spectral response of employed photomultiplier tube. Absorbance (*a*), internal (*η*_IQE_) and external quantum efficiency (*η*_EQE_) of Tb^3+^ PL were obtained through recording all spectra on samples and on a blank reference, using a BaSO_4_-coated integration sphere^20^,^21^.

### Thermal properties

The values of *T*_g_, *T*_c_ and *T*_x_ (peak temperature of crystallization) were obtained from differential scanning calorimetry (DSC, Netzsch DSC 404 F1), using a heating rate of 10 K/min. Non-isothermal crystallization dynamic were studied by DSC (Netzsch DSC 404 F1) on polished bulk glasses (~25–40 mg) at different heating rates of 5, 10, 15 and 20 K/min in order to evaluate the apparent activation energy of crystallization.

### Other properties

The composition of all glasses was verified by wavelength-dispersive electron probe microanalysis (WD-EPMA, microprobe JXA-8800L; Jeol). The ion concentration of Tb^3+^ was calculated according to these compositions. Nominal and as-received compositions are given in Tab. 1. The absence of crystals from the as-made glasses was verified by X-ray diffraction analyses (XRD Siemens Kristalloflex D500, Bragg-Brentano, 30 kV/30 mA, Cu Kα) on bulk samples. The glass density *ρ* was determined in an Archimedes balance, using distilled water as the immersion liquid. The refractive index was determined at the *d* line (n_d_, *λ* = 587 nm) with a Pulfrich reactometer.

## Author Contributions

L.W. and M.S. conceived of the experiment. A.W. prepared all glass samples. G.G., A.W., O.S., J.D. and C.D. performed the experiments. G.G., A.W. and L.W. analyzed the data. G.G. and L.W. wrote the manuscript. All authors contributed to the scientific discussions and manuscript review.

## Figures and Tables

**Table 1:** Nominal and analyzed compositions of the studied glasses (data given in the form “nominal/as-analyzed”, mol%)

Glass sample	GBAG-14Tb	GBAG-18Tb	GBAG-22Tb	GBAG-25Tb
GeO_2_	16.5/17.3	16.5/18.0	16.5/16.4	16.5/17.0
B_2_O_3_	21.5/25.3	21.5/25.0	21.5/21.4	21.5/23.0
Al_2_O_3_	37.0/27.2	37.0/28.0	37.0/37.0	37.0/32.0
Ga_2_O_3_	11.0/15.1	7.0/11.0	3.0/3.2	0.0/0.0
Tb_2_O_3_	14.0/15.1	18.0/18.0	22/22.0	25.0/28.0

**Table 2:** Experimental data of density *ρ*, Tb^3+^ ion concentration, refractive index *n*_d_, Verdet constant *V_B_* (at 632.8 nm), FoM (at 435 nm), *τ*, *T*_g_, *T*_c_, Δ*T* and *E_a_* for the studied glasses

Glass sample	GBAG-14Tb	GBAG-18Tb	GBAG-22Tb	GBAG-25Tb
*ρ*±0.01 (g/cm^3^)	4.08	4.53	4.79	4.85
Tb^3+^ concentration (10^21^ cm^−3^)	5.0	6.5	8.1	9.7
*n*_d @587 nm_ ± 0.01	1.69	1.73	1.74	1.75
*V*_B_ (rad/T/m)	−48	−71	−91	−119
FoM (°/dB)	−0.029	−0.049	−0.023	−0.008
*τ* (ms)	2.23	1.23	0.26	0.10
*T*_g_ ± 1 (°C)	740	767	772	777
*T*_c_ ± 1 (°C)	848	880	905	928
Δ*T* ± 1 (K)	108	113	133	151
*E*_a_ (kJ/mol/K)	520	593	533	482
